# Association between presence of the metabolic syndrome and its components with carotid intima-media thickness and carotid and femoral plaque area: a population study

**DOI:** 10.1186/1758-5996-5-44

**Published:** 2013-08-20

**Authors:** Andrie G Panayiotou, Maura Griffin, Panayiotis Kouis, Theodosis Tyllis, Niki Georgiou, Dawn Bond, Andrew N Nicolaides

**Affiliations:** 1 Cyprus International Institute for Environmental and Public Health in association with Harvard School of Public Health, Cyprus University of Technology, P.O. Box: 50329, Limassol, Cyprus; 2 The Cyprus Cardiovascular Disease Educational and Research Trust, 2 Kyriacou Matsi, Nicosia 2368, Cyprus; 3 Vascular Noninvasive Screening and Diagnostic Centre, 30 Weymouth street, W1G 7BS, London, UK; 4 Vascular Screening and Diagnostic Centre, 2 Kyriacou Matsi, Nicosia 2368, Cyprus; 5 Department of Vascular Surgery, Imperial College, London SW72BX, UK

**Keywords:** Metabolic syndrome, Metabolic syndrome components, Atherosclerotic plaques, Plaque area, Femoral, Carotid, IMT

## Abstract

**Background:**

We aimed to explore the association between presence and number of components of the Metabolic Syndrome (MetS) and subclinical atherosclerosis outcomes (common carotid intima media thickness, plaque presence and sum of plaque area) in both the carotid and femoral bifurcations.

**Methods:**

Cross-sectional analysis of 771 volunteers from the ongoing epidemiological Cyprus Study (46% male; mean age = 60.1 ± 9.8). (a) Carotid intima-media thickness (IMTcc), (b) sum of plaque area in the carotid bifurcations (sum of the largest plaques in each carotid bifurcation-SPAcar), (c) sum of plaque area in the femoral bifurcations (sum of the largest plaques in each femoral bifurcation-SPAfem) and (d) sum of plaque area in both carotid and femoral bifurcations (sum of the areas of the largest plaques present in each of the four bifurcations-SPA) were measured at baseline using ultrasound. Presence and number of components of the MetS was ascertained using the National Cholesterol Education Program ATPIII definition and their association tested using multivariable regression models.

**Results:**

MetS was present in 259 (33.6%) individuals and was associated with a 0.02 mm increase in IMTcc (95% CI: 0.00 to 0.04, p = 0.047) after adjustment for age, sex, family history of CVD, alcohol consumption (BU/week) and smoking (pack-years). Each additional component of the MetS was associated with a 16% higher SPA (95% CI: 6.8% to 25.2%, p_for trend_ = 0.001), a 10% higher SPAcar (95% CI: 5% to 24%, p_for trend_ = 0.003) and a 14% higher SPAfem in the adjusted model.

**Conclusions:**

We confirm an association between the MetS and IMTcc as well as report for the first time an association between the MetS and its components and femoral plaque area, in a general population over 40 years of age. Having any risk factors for the MetS increases the risk for subclinical atherosclerosis, with the risk increasing with each additional component. Using the dichotomous definition of the MetS may be overlooking the risk for subclinical atherosclerosis –and by inference future cardiovascular events- associated with having less than 3 risk factors.

## Background

The Metabolic Syndrome (MetS) currently affects about 25% of the adult population in Europe and this trend is on the rise both in developed but especially in developing countries [1], making it a global public health issue. It is usually defined as a cluster of three or more risk factors for cardiovascular disease (CVD), including hypertension, obesity and dyslipidemia which can identify individuals with increased insulin resistance. Three slightly different definitions are commonly used which result in slightly different estimates of prevalence or incidence of the syndrome: a) the National Cholesterol Education Program III Panel definition (ATPIII), b) the World Health Organization definition and c) the European Group for the Study of Insulin Resistance (EGIR) definition [2], although a “harmonized” definition has also been proposed [3]. Various epidemiological studies have related presence of the MetS with progression of coronary atherosclerosis [4,5], increased risk of cardiovascular disease (CVD) [6,7] and CVD mortality [8,9]. The clinical outcomes of coronary heart disease, CVD and cerebrovascular disease (stroke) are largely attributable to the process of atherosclerosis.

Atherosclerosis can be visualized non-invasively in the arterial wall with the use of high resolution ultrasound and ultrasonic measurements such as intima-media thickness (IMT) are often used as a surrogate end-point in epidemiological studies on CVD and coronary artery disease, with increased carotid IMT (IMTc) having been associated with both the presence and extent of coronary artery disease [10,11], while progression of IMTc has been associated with future cerebrovascular and coronary events [12].

While measuring IMT is becoming common practice, other ultrasonic measurements that include atherosclerotic plaques, such as two dimensional measurements of all the plaques seen in a longitudinal view and the summation of their cross-sectional areas defined as total plaque area (TPA) - provide us with a consistent estimate of the plaque burden [13]. TPA has been found to be associated with increased risk of coronary artery disease and stroke [14,15] and work from us and others [16] has shown that the presence and number of plaques, as well as plaque area, are better predictors of CVD risk compared to common carotid IMT. Although most studies measure carotid plaques only, femoral plaques also predict CVD, independently from carotid plaques [17,18] and plaque area in the femoral arteries may provide additional information on risk.

Previous studies have related presence of the MetS with higher levels of both carotid and femoral IMTc [19-21], higher carotid plaque prevalence [22] and higher total plaque volume [20]. However, only few studies have examined the difference between measures of both carotid and femoral atherosclerosis and the MetS, as well as the loss of informative power when the binary definition of MetS is used instead of the number of MetS components [4,20,22], especially in relation to plaque area.

Therefore, the aim of this study was to explore the association between presence and number of components of the Metabolic Syndrome and subclinical atherosclerosis outcomes (intima media thickness -IMTc, plaque presence and sum of plaque area -SPA) in both the carotid and femoral bifurcations.

## Methods

### Study population

The Cyprus Study is a population-based cohort study of cardiovascular disease and atherosclerosis in 1106 individuals aged 40 years or more from two areas in Cyprus. For description see [23]. Briefly, baseline data have been collected from inhabitants of two randomly selected areas and their relatives who live in any one of the main towns between 2003 and 2008. All inhabitants were identified through the population list held at the Mayor’s office and all those over the age of 40 years were invited to participate. The overall participation rate of those invited was 95%. The Ethics Committee of the Cyprus Institute of Neurology and Genetics approved the study and all participants provided written informed consent. Baseline data from the first 771 subjects with complete data were used in the analysis.

### Cardiovascular disease status, risk factors and ultrasonic measurements

Cardiovascular disease status, risk factors and ultrasonic measurements have been described previously [16]. Briefly, a fasting (6–12 hours) blood sample was obtained for assessment of glucose, insulin, lipid and inflammatory markers.

All scans were performed using a Philips (ATL) HDI 5000 duplex scanner (Seattle, USA). The IMT complex of the far wall of the common carotid artery (IMTcc) was measured at its thickest part (mean of three readings) on both transverse and longitudinal sections 1.5 to 2.0 cm proximal to the bifurcation which was free from any focal thickening i.e. atherosclerotic plaques. All measurements were performed at the time of scanning, with measurements taken 3 times manually in both longitudinal and transverse sections, using the on-screen calipers of the system. No measurements were done remotely. The mean of the measurements from both carotid arteries was used in the analysis. Plaque area was measured off line on a PC by one person using the “Plaque Texture Analysis software” (LifeQMedical Ltd: www.lifeqmedical.com) which is a dedicated research software package. The sum of the areas of the largest plaque in each carotid bifurcation (sum of carotid plaque area: SPAcar), the sum of the areas of the largest plaques in each common femoral bifurcation (sum of femoral plaque area: SPAfem) and the sum of the areas of the largest plaques present in each of the four bifurcations (both carotid and both common femoral) (sum of plaque areas: SPA) were calculated (mm^2^). The two ultra-sonographers who performed the ultrasonic scans were completely blinded to the clinical, biochemical and genetic risk factors of the subjects. The inter-observer mean difference between repeat measurements of IMTcc was −0.03 mm, the within-subject standard deviation was 0.12 mm and the intra-class correlation coefficient was 0.79. For plaque area the corresponding intra-observer values were 0.17 mm^2^, 3.2 mm^2^ and 0.96 mm^2^, respectively.

The National Cholesterol Education Program ATPIII definition was used in this study and diagnosis of MetS was based on subjects exhibiting three or more of the following characteristics: HDL cholesterol <50 mg/dL for women and <40 mg/dL for men; Triglycerides >150 mg/dL; fasting glucose >110 mg/dL; hypertension (>85 mm Hg diastolic pressure and >130 mm Hg systolic pressure) and a waist circumference (WC) of >102 cm for men and > 88 cm for women. In the absence of WC data, a body mass index (BMI) > 30 was used. A “number of components” variable was created according to the number of components present in each individual, ranging from 0 to 5.

#### Statistical analysis

All variables used in the analysis were checked for normality with the use of histograms. Total plaque area was found to be positively skewed and was naturally log-transformed to fit normality. For ln-transformed dependent variables the (B) regression coefficient gives the % change in the dependent for a 1-unit change in the independent variable.

Associations between subclinical atherosclerosis outcomes (IMTcc, SPAfem, SPAcar, SPAtotal) and presence/components of the MetS were investigated by computing age & sex – adjusted basic and multivariable adjusted (age, sex, family history of CVD, alcohol in british units and smoking in pack-years) coefficients using linear regression analysis. Confounders were chosen *a priori*, based on their reported association with prevalence of the MetS [24-26] as well as with the ultrasound traits.

For plaque presence (Yes/No), basic and multivariable adjusted odd ratios (OR) were computed again using binary logistic regression. All analyses were conducted using SPSS Statistics v 18.0 statistical software (SPSS Inc.).

## Results

### Population characteristics

Baseline characteristics of the population are shown in Table 1. Characteristics of the study population according to presence of the MetS are shown in Table 2 for men and women separately. Out of the total 771 included in the analysis, 259 (33.6%) fulfilled the ATP III criteria for MetS (63% men and 37% women). Anti-hypertensive treatment was more frequent in those with the MetS for both sexes (p < 0.001 for both). Women with the MetS were older than those without (p < 0.001) but the same was not true for men (p = 0.17).

**Table 1:** Baseline characteristics of the study population (n = 771) for men and women separately

**Baseline characteristics**	**Men (n = 359)**	**Women (n = 412)**	**P value**
Age (years)*	61.02 (0.56)	60.06 (0.48)	0.19
Systolic blood pressure (mmHg)*	136.47 (0.88)	140.96 (0.86)	<0.001
**BMI (kg/m**^**2**^)*	27.8 (0.22)	28.3(0.25)	0.14
Total Cholesterol (mmol/L)*	5.67 (0.06)	6.0 (0.06)	<0.001
LDL Cholesterol (mmol/L)*	3.47 (0.04)	3.56 (0.037)	0.10
HDL Cholesterol (mmol/L)*	1.16 (0.015)	1.41 (0.015)	<0.001
**Triglycerides (mmol/L)** †	1.55 (1.15; 2.15)	1.40 (1.04; 1.95)	0.002
ApoA1 (g/L)*	1.35 (0.011)	1.53 (0.012)	<0.001
ApoB (g/L)*	1.20 (0.013)	1.19 (0.012)	0.47
**Antihypertensive Treatment (%)**	38.0%	37.0%	0.77
**Antihyperlipidemic Treatment (%)**	21.5%	16.6%	0.08
**Diabetes (%)**	17.3%	9.5%	0.001
**No of MetS components (%)**			
**0 MetS Comp.**	5.3%	12.9%	<0.001
**1 MetS Comp.**	19.6%	35.0%	<0.001
**2 MetS Comp.**	29.1%	28.4%	0.38
**3 MetS Comp.**	25.4%	18.2%	0.21
**4 MetS Comp.**	17.0%	4.4%	<0.001
**5 MetS Comp.**	3.6%	1.2%	0.059

*Mean and standard deviation (sd) is used for normally distributed values.

†Median and interquartile range (iqr) is used for non-normally distributed values.

**Table 2:** Baseline characteristics of the study population by sex, according to presence of the Metabolic syndrome

	**Men (n = 359)**			**Women (n = 412)**		
	**Metabolic syndrome**			**Metabolic syndrome**		
**Baseline characteristics**	**Yes (n = 163)**	**No (n = 196)**	**P value**	**Yes (n = 96)**	**No (n = 316)**	**P value**
Age (years)*	61.86 (±0.83)	60.32 (±0.77)	0.17	64.96 (±0.88)	58.52 (±0.55)	<0.001
Sitting Systolic BP (mmHg)*	141.34 (±1.27)	132.26 (±1.15)	<0.001	151.82 (±1.16)	137.49 (±0.96)	<0.001
Sitting Diastolic BP (mmHg)*	85.69 (±0.77)	80.79 (±0.73)	<0.001	89.27 (±0.92)	82.67 (±0.51)	<0.001
**Antihypertensive Therapy (%)**	50.9%	28.4%	<0.001	60.4%	29.4%	<0.001
Total Cholesterol (mmol/L)*	5.61 (±0.08)	5.70 (±0.08)	0.56	6.04 (±0.1)	5.99 (±0.06)	0.67
LDL cholesterol (mmol/L)*	3.44 (±0.06)	3.49 (±0.06)	0.52	3.58 (± 0.06)	3.54 (± 0.04)	0.66
HDL cholesterol (mmol/L)*	1.03 (±0.63)	1.27 (±0.02)	<0.001	1.20 (± 0.02)	1.48 (±0.016)	<0.001
**Triglycerides (mmol/L)†**	2.10 (1.63; 2.64)	1.32 (0.98; 1.57)	<0.001	2.15 (1.78; 2.83)	1.24 (0.97; 1.56)	<0.001
**Diabetes mellitus (%)**	30.7%	6.3%	<0.001	33.3%	2.3%	<0.001
**BMI (kg/m**^**2**^)*	29.88 (±0.35)	26.06 (±0.2)	<0.001	32.39 (±0.47)	26.95 (±0.25)	<0.001
ApoA1 (g/L)*	1.29 (±0.013)	1.40 (±0.017)	<0.001	1.44 (±0.02)	1.55 (±0.001)	<0.001
ApoB (g/L)*	1.21 (±0.02)	1.19 (±0.02)	0.48	1.23 (±0.02)	1.18 (±0.014)	0.044
**Homa index†**	2.25(1.15; 4.18)	0.94(0.51; 1.8)	<0.001	2.51 (1.67; 3.86)	1.14 (0.72; 1.7)	<0.001
IMTccMean (mm)*	0.79 (±0.15)	0.76 (±0.12)	0.084	0.77 (±0.16)	0.69 (±0.13)	<0.001
**Sum of total plaque area (mm**^**2**^**)†**	53 (25; 103)	42 (10;72)	0.002	16 (1; 44)	8 (1;32)	0.01
**Sum of plaque area carotids (mm**^**2**^**)†**	22 (1; 39)	12 (1; 27.5)	0.011	8.75 (1;25.75)	1 (1; 19)	0.013
**Sum of plaque area femoral (mm**^**2**^**)†**	33(10; 62)	23(1; 49)	0.011	1(1; 17.75)	1(1; 6)	0.012

*Mean and standard deviation (sd) is used for normally distributed values.

†Median and interquartile range (iqr) is used for non-normally distributed values.

### IMTcc

**Figure 1:**
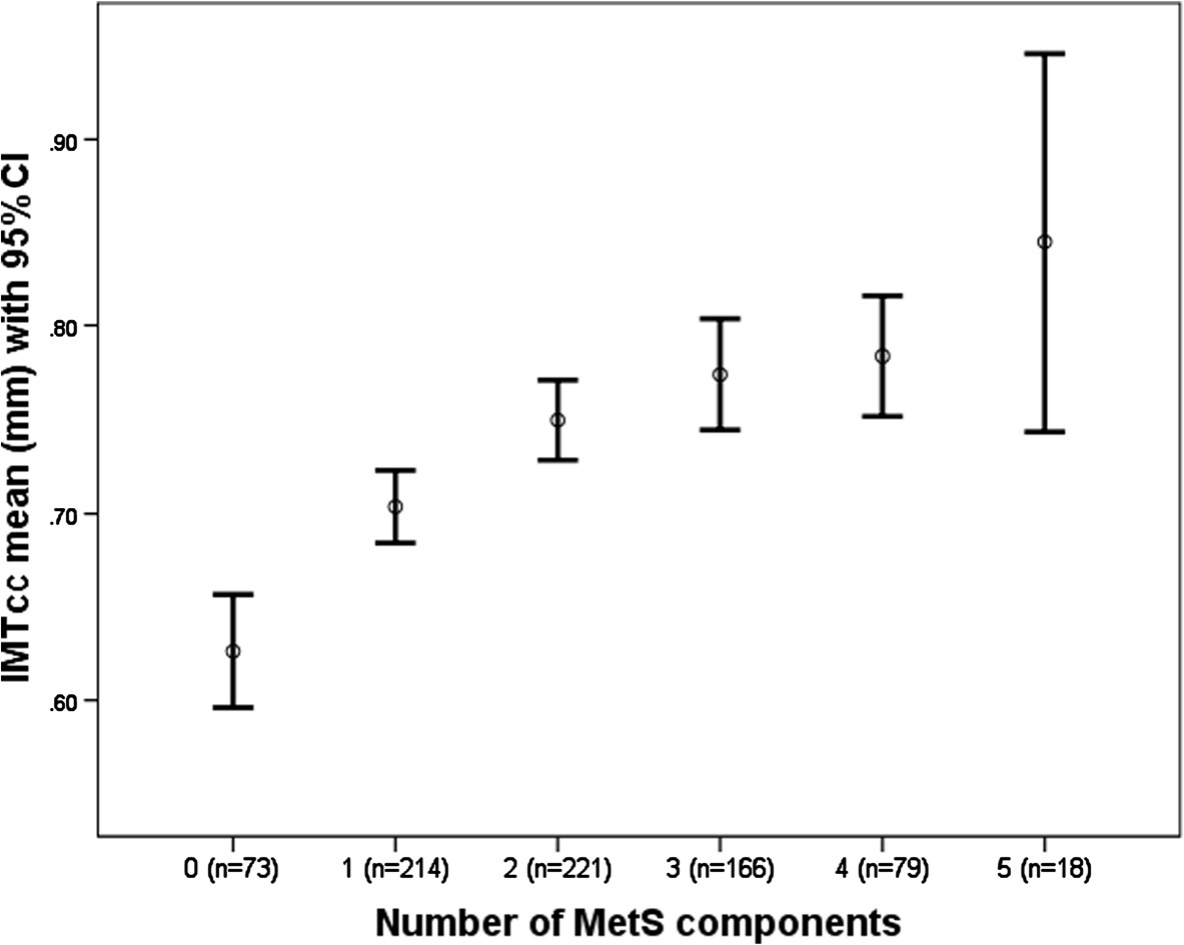
Association between the number of MetS components and IMTcc.

In our study population, IMTcc was found to be higher in those with the MetS, corresponding to a 0.03 mm (95% CI: 0.00 to 0.05, p = 0.02) increase in mean IMTcc in the basic model and a 0.02 mm (95% CI: 0.00 to 0.04, p = 0.047) increase in the fully adjusted model for those with the MetS. When the number of components of the Mets was used in the analysis, each additional component was found to be associated with a 0.02 mm (95% CI: 0.01 to 0.03, p_for trend_ <0.001) increase in the basic model and a 0.02 mm increase in the fully adjusted model (95% CI: 0.01 to 0.02, p_for trend_ < 0.001). Both the p value for trend obtained from the model and a visual inspection of the data (Figure 1), suggested that the association between IMTcc and the number of components was linear and therefore agreed with the effect being additive. Results are shown in detail in Table 3.

**Table 3:** Associations between (i) metabolic syndrome presence and (ii) number of components (exposures) and atherosclerosis outcomes

	Basic model*						**Adjusted model†**					
	**(i)Metabolic syndrome**			**(ii)Number of components present**			**(i)Metabolic syndrome**			**(ii)Number of components present**		
	**B (95% CI)**	**P**	**R**^**2**^	**B (95% CI)**	**P**^**#**^	**R**^**2**^	**B (95% CI)**	**P**	**R**^**2**^	**B (95% CI)**	**P**^**#**^	**R**^**2**^
IMTcc (mm)	0.03 (0.00 to 0.05)	0.02	0.278	0.02 (0.01 to 0.03)	<0.001	0.286	0.02 (0.00 to 0.04)	0.047	0.287	0.02 (0.01 to 0.02)	<0.001	0.296
(ln)SPA	0.24 (0.002 to 0.47)	0.048	0.330	0.17 (0.07 to 0.26)	<0.001	0.337	0.22 (−0.015 to 0.45)	0.07	0.344	0.16 (0.07 to 0.25)	0.001	0.351
(ln)SPAcar	0.15 (−0.07 to 0.38)	0.18	0.234	0.10 (0.01 to 0.19)	0.03	0.237	0.16 (−0.07 to 0.38)	0.17	0.241	0.10 (0.01 to 0.19)	0.03	0.244
(ln)SPAfem	0.26 (0.02 to 0.50)	0.03	0.303	0.15 (0.05 to 0.24)	0.003	0.307	0.24 (0.004 to 0.48)	0.046	0.333	0.14 (0.05 to 0.24)	0.003	0.337
	**OR (95% CI)**	**P**	**R**^**2**^	**OR (95% CI)**	**P**	**R**^**2**^	**OR (95% CI)**	**P**	**R**^**2**^	**OR (95% CI)**	**P**	**R**^**2**^
Plaque Presence (0 Vs 1–4)	1.42 (0.93 to 2.16)	0.10	0.303	1.27 (1.08 to 1.5)	<0.001	0.319	1.38 (0.91 to 2.12)	0.13	0.312	1.25 (1.06 to 1.48)	0.008	0.326

*Basic model: adjusted for age and sex.

†Adjusted model: adjusted for age, sex, family history of CVD, alcohol consumption (BU/week) and smoking (pack-years).

# P for trend.

For ln-transformed outcomes of TPA, the B coefficient gives the% change per unit change in the independent.

R^2^: adjusted R^2^ for linear regression and Nagelkerke pseudo-R^2^ for logistic regression models.

P value for model fit (F statistic) <0.001 for all models.

### Sum of total plaque area

**Figure 2:**
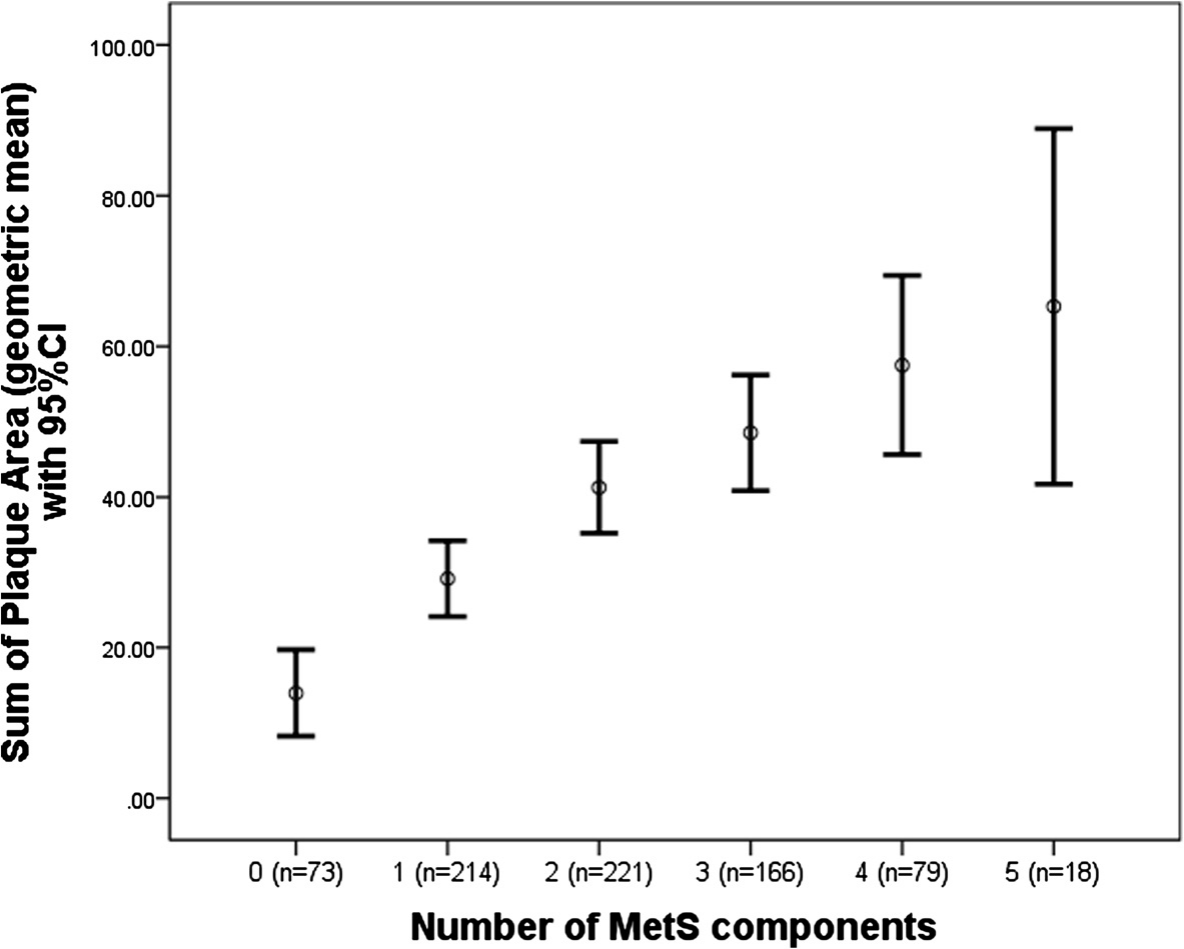
Association between the number of MetS components and sum of plaque area (SPA).

Presence of the MetS was found to be associated with a 24% (95% CI: 0.2% to 47%, p = 0.048) increase in total plaque area (SPA) in the basic model. After further adjustment, the effect was only slightly attenuated but it was no longer conventionally statistically significant (OR_adjusted_ = 22%; 95% CI: -1.5% to 45%, p = 0.07). When the number of MetS components was examined, these were significantly associated with increased SPA estimates, both in the basic and the fully adjusted model (Table 3). Each MetS Component was associated with a 17% increase in SPA in the basic model (95% CI: 7% to 26%, p_for trend_ < 0.001) and with a 16% increase in the adjusted model (95% CI: 7% to 25%, p_for trend_ = 0.001), indicating minimal confounding by family history of CVD, alcohol and smoking and suggestive again of a linear association (Figure 2).

### Carotid and femoral plaque area

When looking at the site of plaque, presence of the MetS was found to be associated with SPA in the femoral but not with SPA in the carotid arteries after adjustment (OR_adjusted_ = 24%; 95% CI: 0.4% to 48%, p = 0.046 and OR_adjusted_ = 16%; 95% CI: -7% to 38%, p = 0.17 respectively).

When looking at the number of MetS components, (an arguably more informative exposure), these were significantly associated with increased plaque area in both the femoral and carotid bifurcations, suggesting that the lack of association seen previously with SPAcar may have been a spurious finding, althoughthe effect size was somewhat larger for plaque area in the femoral arteries. Each additional MetS component was associated with a 14% higher SPAfem (95% CI: 5% to 24%, p_for trend_ = 0.003) and a 10% higher SPAcar (95% CI: 1% to 19%, p_for trend_ = 0.031) in the fully adjusted model.

### Plaque presence

Presence of the MetS was not statistically significantly associated with the number of plaques present (0 Vs 1–4) in our study population (OR_basic_ = 1.42; 95% CI: 0.93 to 2.16, p = 0.10 and OR_adjusted_ = 1.38; 95% CI: 0.91 to 2.12, p = 0.13). When looking at the number of individual components of the Mets, these were found to be strongly associated with the odds of having a plaque with each additional component being associated with ≈ 25% increased odds for plaque in the fully adjusted model (OR _adjusted_ =1.25; 95% CI: 1.06 to 1.48, p_for trend_ = 0.008), suggesting that the risk for atherosclerotic plaques increases before reaching the cut-off point of ≥3 risk factors for diagnosis of the MetS.

## Discussion

In this population-based study of middle aged men and women, we show that the MetS was associated with measures of subclinical atherosclerosis, including sum of total plaque area in the femoral arteries, and that the number of components of the MetS was better than the binary diagnosis of the MetS in predicting subclinical atherosclerosis. The association was best fitted by a linear model, were even one component of the MetS was associated with increased IMTcc and SPA (Figures 1 and 2).

A number of studies have shown both presence of the MetS [20,21,27,28], and the number of components [20,29-32], to be significantly associated with IMTc [20,21,27,28] as well as provide evidence for a linear association, with increasing number of MetS components being associated with increased IMTc [20,29-32]. We also show an increase of 0.17 mm in IMTc between those with 0 Vs those with ≥4 components as previously reported by Adolphe et al. [29], with the biggest increase in our population seen between 0 and 1 components (0.08 mm) (Figure 1). Additionally we report an increase in IMTcc of 0.02 mm for every additional component (p_for trend_ < 0.001) after adjustment for age, sex, family history of CVD, smoking and alcohol. Although the time frame associated with the 0.02 mm increase is not known due to the cross-sectional nature of the study, a 0.03 mm/yr increase in IMTcc has been previously shown to be associated with 2.2 times increase in risk for coronary events [33].

When stratifying by sex, the unadjusted association between IMTcc and MetS presence was more pronounced in women (Table 2). This is in agreement with previously published studies both for baseline IMTc [32,34,35] and annual IMTc change [36], pointing to a possible interaction between sex and the MetS for IMTc, although reports have been published both for [32,34] and against [31] such an interaction. In our population there was no statistically significant interaction between sex and the MetS for IMTcc (p_for interaction_ = 0.46) and we therefore analysed the population as a whole.

Total plaque area is now emerging as a better predictor of atherosclerotic disease and cardiovascular risk as compared to IMTcc [37,38], with researchers from the Trømso study reporting that the association between IMTc and myocardial infarction (MI), as well as first ischemic stroke [39] was weaker than between total plaque area and MI [40]. Adding to this, a recent meta-analysis has shown that assessment of carotid plaque had a higher diagnostic accuracy for the prediction of future coronary artery disease events compared with that of carotid IMT [41]. We report a 25% higher risk of having any plaque present in both carotid and femoral arteries with each additional component (OR = 1.25, 95% CI:1.06 to 1.48, P_for trend_ = 0.008), indicative of a linear association. When looking at plaque area, we show a weak association between presence of the MetS and sum of plaque area in all four bifurcations (SPA), but a much stronger association when using the number of components variable; with each additional component of the MetS being associated with a 16% higher SPA (p_for trend_ = 0.001), again indicative of a linear association (Figure 2). Those with 0 components had a median SPA of 0 (i.e.no plaques), compared to a median SPA of 17 mm^2^ for 1 component and a SPA of 41 mm^2^ for ≥4 components (Figure 2).

Our results are in agreement with the only other study reporting on plaque presence (albeit only in the carotids) and components of the MetS in a multiethnic population [22], which showed a significant increase in the ORs for each additional component, both for carotid plaque presence and carotid plaque thickness. A smaller study in a homogeneous Indian population supported a similar, although not statistical significant trend for Total Plaque Volume (TPV) [20]. Similar findings have been reported for plaque progression (>5% increase in percent atheroma volume), concluding that individual components are the driving force behind the association between MetS and progression of atherosclerosis and not the binary presence of the syndrome itself [4].

To the best of our knowledge, this is the first report of an association between the MetS and number of components and sum of plaque area in the common femoral arteries. We report a strong association between the number of components of the MetS and SPA in both the carotid and femoral bifurcations, with the magnitude of the association being somewhat greater for the femoral bifurcations (10% Vs 14%) [42,43].

In our study, the number of components of the MetS was more strongly associated with measures of subclinical atherosclerosis compared to the diagnosis of the MetS alone, and supported a linear association with both IMTcc and SPA, with each additional component being associated with a higher IMTcc and SPA. Although current definitions of the MetS do not differentiate between components, when adding all five individual components in a step-wise regression model (adjusting for age and sex), hypertension was the only one left in the model for IMTcc, SPA and SPAcar (B = 0.04, B = 0.41 and B = 0.38 respectively; p < 0.001 for all). When looking at SPAfem, out of the five components, only hypertriglyceridemia was left in the model (B = 0.31, p = 0.008), reflecting perhaps the different pathophysiology between arterial beds in support of site-related differences in atherosclerotic plaque development [44] and progression [43]. Our results are in agreement with recent reports from the Trømso study, that hypertension was the only MetS component consistently associated with both levels of IMTc and total plaque area in the carotids [32].

Although not directly comparable, reports for CVD and T2DM [45,46], as well as compromised structure and function of the heart [47] and coronary heart disease [48] further support our results, showing loss of information when using the binary presence of the MetS definition. In a diabetic population, Sone H. et al., [49] estimated a significantly elevated risk for CVD in subjects with at least two metabolic components present and a very recent study reported that the clinical usefulness of the MetS for risk of CHD did not exceed the sum of its individual components, as after adjustment for its components, MetS was no longer associated with the disease [50]. On the other hand, another recent study stated that the number of MetS components was only as informative as the (dichotomous) MetS classification in the prediction of CVD in a Chinese population [51].

Whether or not use of the MetS definition is justified when estimating risk for CVD, as reviewed comprehensively by Reaven et al. [52], it is still being used by clinicians. It is therefore important to understand that when using the MetS dichotomous definition one may be overlooking the increase in risk for atherosclerosis and CVD associated with having less than 3 risk factors.

Our study is not without its limitation. As mentioned earlier this is a cross-sectional analysis so we cannot make any causal inferences about the role of the MetS components on subclinical atherosclerosis outcomes. In the majority of the cases, waist circumference measurements were not available and BMI was used instead. The WC cut-off points used though are in agreement with the National Institutes of Health obesity guidelines, which equate to a BMI of ~30 Kg/m^2^ in men [53]. Additionally, we used the ATPIII definition for the MetS, which is slightly different from the “Harmonised” definition currently proposed [3]. The only difference between the two definitions though is the cut-off point for fasting glucose (>110 mg/dL in the ATPIII Vs >100 mg/dL in the “harmonized”), which is not expected to have influenced our results.

## Conclusion

In conclusion, we confirm an association between the MetS and both IMTcc and carotid plaques, as well as report for the first time an association between the MetS and its components and femoral plaque area, in a general population over 40 years of age. Having any risk factors for the MetS increases the risk for subclinical atherosclerosis, with each additional component contributing to the risk. Using the dichotomous definition of the MetS may be overlooking the risk for subclinical atherosclerosis –and by inference future cardiovascular events- associated with having less than 3 risk factors.

## Abbreviations

MetS: Metabolic syndrome; CVD: Cardiovascular disease; ATPIII: National cholesterol education program III; EGIR: European group for the study of insulin resistance; cIMT: Carotid intima media thickness; TPA: Total plaque area; SPAcar: Sum of carotid plaque area; SPAfem: Sum of femoral plaque area; SPA: Sum of plaque areas; WC: Waist circumference; BMI: Body mass index; MI: Myocardial infarction; TPV: Total plaque volume; T2DM: Type 2 diabetes mellitus.

## Competing interests

The authors declare no competing interests.

## Authors’ contributions

AP participated in the design of the study, performed the biochemical analyses and statistical analysis and drafted the manuscript. MG performed the ultrasonic analysis and plaque area analysis. PK performed the statistical analysis and helped draft the manuscript. TT performed ascertainment and analysis of clinical and metabolic syndrome risk factors. NG performed ultrasonic and IMT analysis. DB performed the co-ordination of the study. AN conceived the study, participated in its design and plaque area analysis and commented on the manuscript. All authors read and approved the final manuscript.
